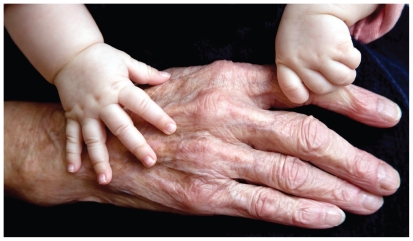# Breaking Patterns of Disease: Early-Life Clues May Predict Long-Term Health

**DOI:** 10.1289/ehp.118-2920107

**Published:** 2010-08

**Authors:** M. Nathaniel Mead

**Affiliations:** **M. Nathaniel Mead**, a science writer living in Durham, NC, has written for *EHP* since 2002

Modern diseases often seem to occur in isolation, but many are now known to emerge from a complex web or pattern of conditions linked together by certain underlying biological mechanisms and processes. With the help of large disease databases, medical scientists have begun to discern how such patterns occur over the course of a lifetime. A new review focused on developmental immunotoxicology explores how this integrative perspective might inspire novel strategies for lowering the risk and prevalence of immune-based diseases influenced by environmental stimuli **[*EHP*** **118(8):1091–1099; Dietert et al.]**.

Many chronic diseases share three common features: 1) early-life exposures to chemical agents or pathogens, 2) evidence of immune insult or dysfunction, and 3) the appearance of disease biomarkers in exposed children although disease itself may not manifest until later in life. One example of interlinked disease conditions highlighted by the authors is metabolic syndrome, defined as the co-occurrence of at least three of five conditions: insulin resistance, obesity, high blood pressure, elevated triglycerides, and reduced HDL cholesterol.

Immune dysfunction is central to the underlying physiology of metabolic syndrome, and the authors posit that the seeds of such dysfunction may be planted in childhood. They describe pre- and postnatal exposures to environmental risk factors that produce postnatal lipid dysregulation and immune dysfunction. However, it is not yet known whether immune dysfunction is an underlying cause of metabolic syndrome or simply an associated or disease-facilitating characteristic.

The practical key to preventing metabolic syndrome may lie in treatments that address overall patterns and their progression, not just the initial presenting condition. “For those patterns of disease with immune involvement,” the authors write, “preventing the underlying immune dysfunction is the single most effective option to minimize the risk of one or more chronic diseases later in life.” This will require more information about risk factors for immune dysfunction that are encountered during development or childhood. Therefore, the authors also recommend that chemicals and pharmaceuticals be tested for developmental immunotoxicity end points; currently, safety assessments are based solely on adult exposures.

The authors say patterns of disease can be used to better predict, prevent, and treat diseases associated with an immune-related pattern of diseases, and may also serve as the basis for environmental protection and testing to prevent exposure to developmental immunotoxicants that may contribute to multiple interconnected diseases. But pattern-based evaluation, prevention, and treatment will require a shift from the prevailing single-organ approach to disease classification and management.

## Figures and Tables

**Figure:**